# The impact of preoperative 5-alpha reductase inhibitors on functional outcomes and health-related quality of life following radical prostatectomy – A propensity score matched longitudinal study

**DOI:** 10.1007/s00345-024-05108-9

**Published:** 2024-07-22

**Authors:** Thilo Westhofen, Kerstin Frank, Alexander Buchner, Armin Becker, Lennert Eismann, Severin Rodler, Can Aydogdu, Elena Berg, Friedrich Jokisch, Philipp M. Kazmierczak, Christian G. Stief, Alexander Kretschmer

**Affiliations:** 1 Department of Urology, Ludwig-Maximilians-University of Munich, Marchioninistrasse 15, 81377 Munich, Germany; 2 Department of Radiology, Ludwig-Maximilians-University of Munich, Munich, Germany; 3 Janssen Oncology Research and Development, Los Angeles, CA USA

**Keywords:** Radical prostatectomy, Prostate cancer, HRQOL, EORTC QLQ-C30, health-related quality of life

## Abstract

**Objectives:**

While the impact of treatment with 5-alpha Reductase Inhibitors (5-ARI) on the risk of cancer-related mortality in men with prostate cancer (PC) has been extensively studied, little is known about the impact of preoperative 5-ARI use on patient-reported outcomes (PROs) following radical prostatectomy (RP).

**Methods:**

Within our prospectively maintained institutional database of 5899 patients treated with RP for PC (2008– 2021), 99 patients with preoperative 5-ARI therapy were identified. A 1:4 propensity-score matched analysis of 442 men (*n* = 90 5-ARI, *n* = 352 no 5-ARI) was conducted. Primary endpoint was continence recovery using daily pad usage and ICIQ-SF. Health-related quality of life (HRQOL) was assessed using the validated EORTC QLQ-C30 and PR25 questionnaires. Multivariable Cox-regression-models tested the effect of preoperative 5-ARI treatment on continence-recovery (*p* < 0.05).

**Results:**

Patients were followed up perioperatively, followed by annual assessments up to 60mo postoperatively. Preoperative mean ICIQ-SF score (2.2 vs. 0.9) was significantly higher in the 5-ARI cohort (*p* = 0.006). 24mo postoperatively, 68.6% (no 5-ARI) vs. 55.7% (5-ARI) had full continence recovery (*p* = 0.002). Multivariable Cox regression analysis, revealed preoperative 5-ARI treatment as an independent predictor for impaired continence recovery (HR 0.50, 95% CI 0.27–0.94, *p* = 0.03) In line, general HRQOL was significantly higher for patients without 5-ARI only up to 24mo postoperatively (70.6 vs. 61.2, *p* = 0.045). There was no significant impact of preoperative 5-ARI treatment on erectile function, biochemical recurrence-free survival and metastasis-free survival.

**Conclusions:**

Pre-RP 5-ARI treatment was associated with impaired continence outcomes starting 24mo postoperatively, suggesting that preoperative 5-ARI treatment can impair the long-term urinary function recovery following RP.

**Supplementary Information:**

The online version contains supplementary material available at 10.1007/s00345-024-05108-9.

## Introduction

5-alpha reductase inhibitors (5-ARI) are frequently used for the treatment of benign prostatic hyperplasia (BPH) due to their ability to reduce prostate size through blockade of the enzymatic conversion of testosterone into its biologically active form, dihydrotestosterone [1]. Based on this mode-of-action, the impact of 5-ARI on variable prostate cancer (PCa) outcomes has been extensively studied. In a large population-based study, Björnebo et al. did not find an association of 5-ARI use with prostate cancer mortality in men without previous PCa diagnosis [2]. For men previously diagnosed with low-risk PCa, it has been shown that long-term use of 5-ARI is safe during active surveillance and does not increase the risk of upstaging to high-risk disease [3]. Post-radical prostatectomy (RP), no difference in the rate of adverse pathology findings between 5-ARI users and nonusers could be detected in a large active surveillance cohort [4]. In contrast to the evidence that has been gathered regarding the effect of 5-ARI use on cancer-specific outcomes, the impact on post-RP functional outcomes is still poorly understood. Furthermore, the effect on Health-related quality of life (HRQOL) remains unknown. Driven by this paucity of data, we conducted this first propensity-score (PS)-matched analysis of a large contemporary cohort of patients who underwent RP for PCa with or without 5-ARI therapy prior surgery. Hereby, we tested the hypothesis that preoperative 5-ARI use impacts patient-reported outcomes in patients following RP.

## Materials and methods

### Patient population, study design and data assessment

Following approval by a local ethics committee (#20-1022), 6825 patients from a prospectively maintained institutional database who underwent RP for PCa between January 2008 and September 2021 were identified. Surgical techniques in our department have been described previously [5]. 5899 patients met the inclusion criteria of the study which encompassed: surgery performed by high-volume surgeons with more than 200 previous RP. Exclusion criteria were: Patients with pT4-disease (*n* = 18), preoperative indicative for metastatic disease (*n* = 301), neoadjuvant treatment prior RP (*n* = 102) and patients with incomplete data or lost to follow-up (*n* = 472) (suppl. Figure 1). 99 patients were identified with 5ARI-use prior RP, defined as a documented treatment with 5-ARIs at the time of diagnosis of prostate cancer. PS-matching limited to eligible patients was carried out applying matching variables: age, BMI, pT-stage, Gleason grade, positive surgical margin rate and rate of robot-assisted laparoscopic RPs (RALP). PS-matching was conducted in a 1:4 manner, applying nearest neighbour matching with a matching tolerance of 0.0001, resulting in a matched cohort of 442 patients [*n* = 90 patients with preoperative 5-ARI treatment (5ARI), *n* = 352 patients with without preoperative 5-ARI treatment (no 5ARI)]. The ratio of PS-matching was set at 1:4 in order to reduce selection bias of PS-matching [6].

### Outcomes

Primary endpoint was continence recovery. Urinary continence was assessed by the International Consultation of Urinary Incontinence questionnaire in its short-form (ICIQ-SF) [7], and daily pad usage. Continence recovery was defined by use of up to one security pad per 24 h. Secondary endpoint was HRQOL. Prospective assessment of HRQOL was performed using a validated translation of the standardised European Organization for Research and Treatment of Cancer (EORTC) quality of life questionnaire (QLQ)-C30 and its prostate specific QLQ-PR25 add-on [8]. According to established cut-off values, “good general HRQOL” was defined as a global health status (GHS) of ≥70 [9]. Erectile function was assessed with the simplified International Index on Erectile Function (IIEF-5) questionnaire [10]. As per institutional standard of care, questionnaires were handed out to patients 1 to 3 days prior to RP. Further endpoints were biochemical recurrence-free survival (BRFS), and metastasis-free survival (MFS) based on conventional or PET-based imaging, which were calculated from date of the radical prostatectomy (RP). Patients were censored at last follow-up including imaging or death.

### Follow up

Follow-up of eligible patients was performed at 3-month intervals within the first postoperative year, followed by annually intervals thereafter. Validated questionnaires were sent via mail to eligible patients. In addition, oncological outcome information was retrieved directly from patients, referring urologists, and primary physicians.

### Statistical analysis

Statistical analyses and reporting and interpretation of the results were conducted according to Guidelines for Reporting of Statistics for Clinical Research in Urology [11]. For descriptive statistics, median and means were used to present continuous variables and percentages or absolute numbers to present non-continuous variables. Separate longitudinal modelling of ICIQ-SF-scores and general HRQOL stratified by preoperative 5-ARI usage was performed.

Continence recovery and survival probabilities of subgroups stratified by preoperative 5-ARI usage were estimated applying Kaplan-Meier method and compared using log-rank test. Multivariable Cox-regression models were used to examine the independent prognostic impact of preoperative 5-ARI use on continence recovery, stratified by sociodemographic and clinicopathological variables, which have previously shown to be relevant confounders [12, 13]. A *p*-value of < 0.05 was considered statistically significant. Statistical analysis was performed using MedCalc Statistical Software version 20.011 (MedCalc Software, Belgium).

## Results

### Perioperative patient characteristics

Patient characteristics of the unmatched and matched cohorts are displayed in Table 1. Applying PS-matching, a well-balanced cohort of 442 patients was generated [*n* = 90 (5-ARI), *n* = 352 (no 5-ARI)]. Median follow-up was 45 months. In the matched cohort, median preoperative PSA level was significantly lower in the 5-ARI subcohort (6.9 vs. 8.2ng/dl, *p* = 0.031). In addition, the median IPSS score was significantly higher for the no-5-ARI cohort (8 vs. 11, *p* = 0.024). All other baseline parameters were well-balanced between both subcohorts (p-range: 0.322–0.942).

**Table 1:** Baseline characteristics of the unmatched and matched cohorts included in the current study (BMI = body-mass index, IPSS = international prostate symptom score, IQR = interquartile range, PSA = prostate-specific antigen, RALP = robot-assisted laparoscopic radical prostatectomy, RP = radical prostatectomy). Bold values indicate *p* < 0.05

	unmatched cohort			matched cohort		
	5ARI	no 5ARI	*p*	5ARI	no 5ARI	*p*
No. of patients	99	5800		90	352	
Age, yrs [median, IQR]#	72 [65,76]	66 [60,71]	***< 0.001***	72 [65,76]	71.5 [66,75]	0.811
BMI kg/m2 [median, IQR]#	26.6 [24.5,29.1]	26.3 [24.3,28.7]	0.647	26.6 [24.4,29.2]	26.7 [24.7,29.7]	0.539
PSA preop. ng/ml [median, IQR]	6.9 [4,12]	8 [5.5,13.4]	***0.007***	6.9 [4.0,11.9]	8.2 [5.5,12.0]	***0.031***
Prostate volume ml [median, IQR]	61.5 [45.8,78]	52 [42,66]	***< 0.001***	60 [45,76]	58 [45,73]	0.527
IPSS [median, IQR]	7 [3,14]	11 [5,15]	***0.007***	72 [65,76]	71.5 [66,75]	0.811
Gleason score [n (%)]#						
6	11 (11.2)	1131 (19.5)	***0.003***	11 (12.2)	48 (13.6)	0.919
7a	21 (21.2)	1908 (32.9)		20 (22.2)	83 (23.6)	
7b	32 (32.3)	1091 (18.8)		28 (31.1)	108 (30.7)	
8	11 (11.1)	696 (12.0)		9 (10.1)	37 (10.5)	
9	22 (22.2)	893 (15.4)		20 (22.2)	73 (20.7)	
10	2 (2.0)	81 (1.4)		2 (2.2)	3 (0.9)	
pT stage [n (%)]#						
pT2a	10 (10.1)	400 (6.9)	0.171	7 (7.8)	26 (7.4)	0.942
pT2b	3 (3.0)	128 (2.2)		3 (3.3)	10 (2.8)	
pT2c	40 (40.4)	2813 (48.5)		37 (41.1)	164 (46.6)	
pT3a	17 (17.2)	1224 (21.1)		16 (17.8)	63 (17.9)	
pT3b	29 (29.3)	1235 (21.3)		27 (30)	89 (25.3)	
Positive surgical margin [n (%)]#	18 (18.2)	1363 (23.5)	0.279	16 (17.8)	81 (23.0)	0.322
Lymph node involvement [n (%)]	11 (11.1)	621 (10.7)	0.898	11 (12.2)	47 (13.4)	0.777
post RP radiotherapy [n (%)]	34 (34.3)	2514 (43.3)	0.082	28 (31.1)	121 (34.4)	0.618
PSA persistance [n (%)]	26 (26.3)	911 (15.7)	***0.009***	19 (21.1)	52 (14.8)	0.150
Nerve-sparing [n (%)]	87 (87.9)	5052 (87.1)	0.974	80 (88.9)	298 (84.7)	0.352
Robot assisted RP [n (%)]#	38 (38.4)	1314 (22.7)	***< 0.001***	34 (37.8)	126 (35.8)	0.727
post RP pelvic floor muscle training [n (%)]	91 (91.9)	5469 (94.3)	***0.758***	83 (92.2)	328 (93.2)	0.510

# propensity score matched variables

### Preoperative 5-ARI use and postoperative continence recovery

Preoperative as well as postoperative comparison of continence outcomes based on daily pad usage and ICIQ-SF scores are summarized in suppl. Table 1.

Based on the validated ICIQ-SF questionnaire, significantly lower ICIQ-SF scores were detected for the no-5-ARI subgroup, indicating better continence outcomes at this time-point.

**Fig. 1:**
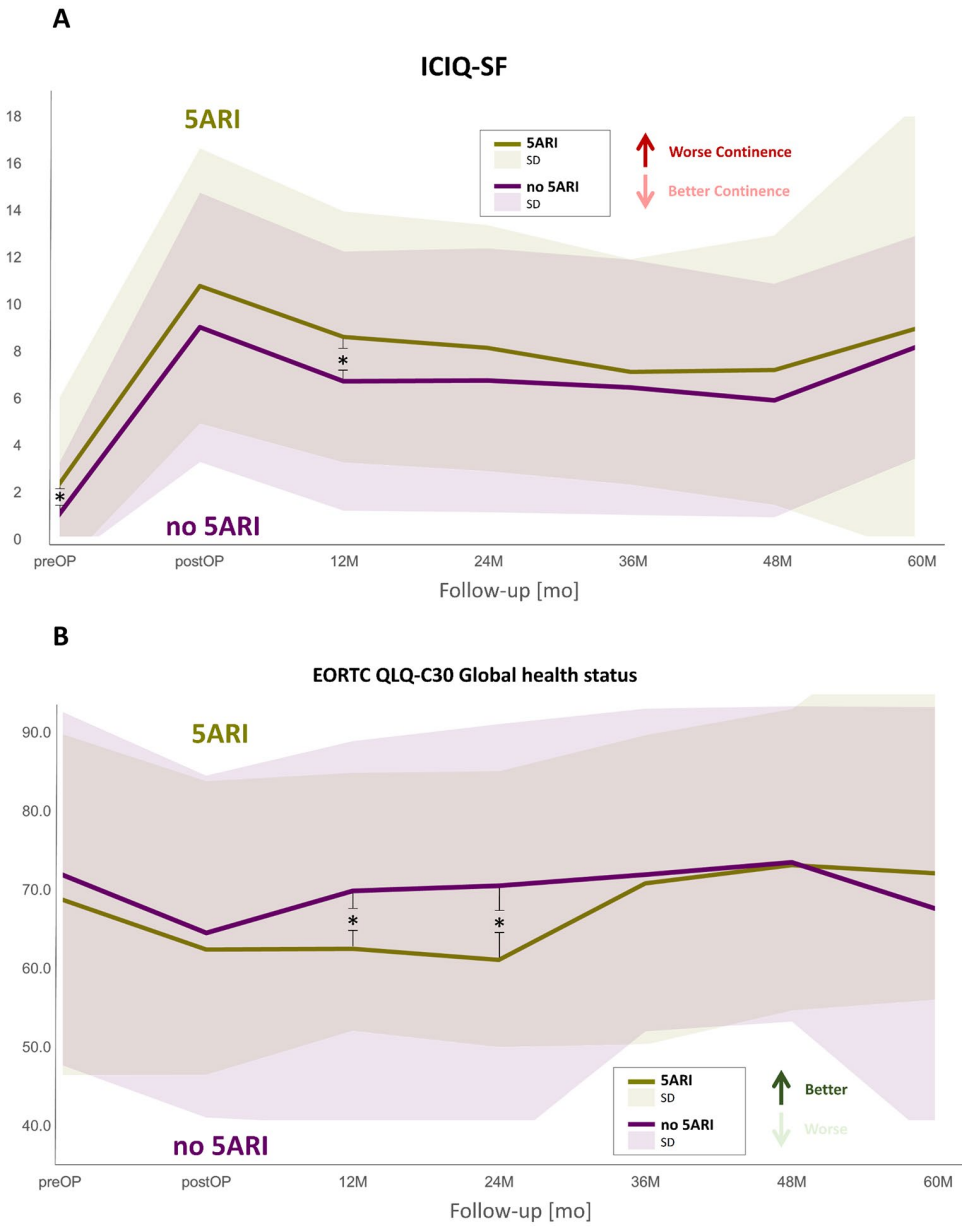
Longitudinal assessment of the mean (**A**) ICIQ-SF-score and (**B**) EORTC QLQ-C30 Global health status stratified by preoperative 5-ARI usage [*p < 0.05]

Up to 12 months postoperatively, continence recovery rates were numerically higher in the no-5-ARI subgroup without reaching statistical significance. Starting at 24 months postoperatively, significantly higher continence recovery rates were detected for the no-5-ARI patients throughout the post-treatment follow-up phase, reaching continence recovery rates of 67.5 vs. 52.9% (*p* = 0.020) 60 months postoperatively (Fig. 1A).

**Fig. 2:**
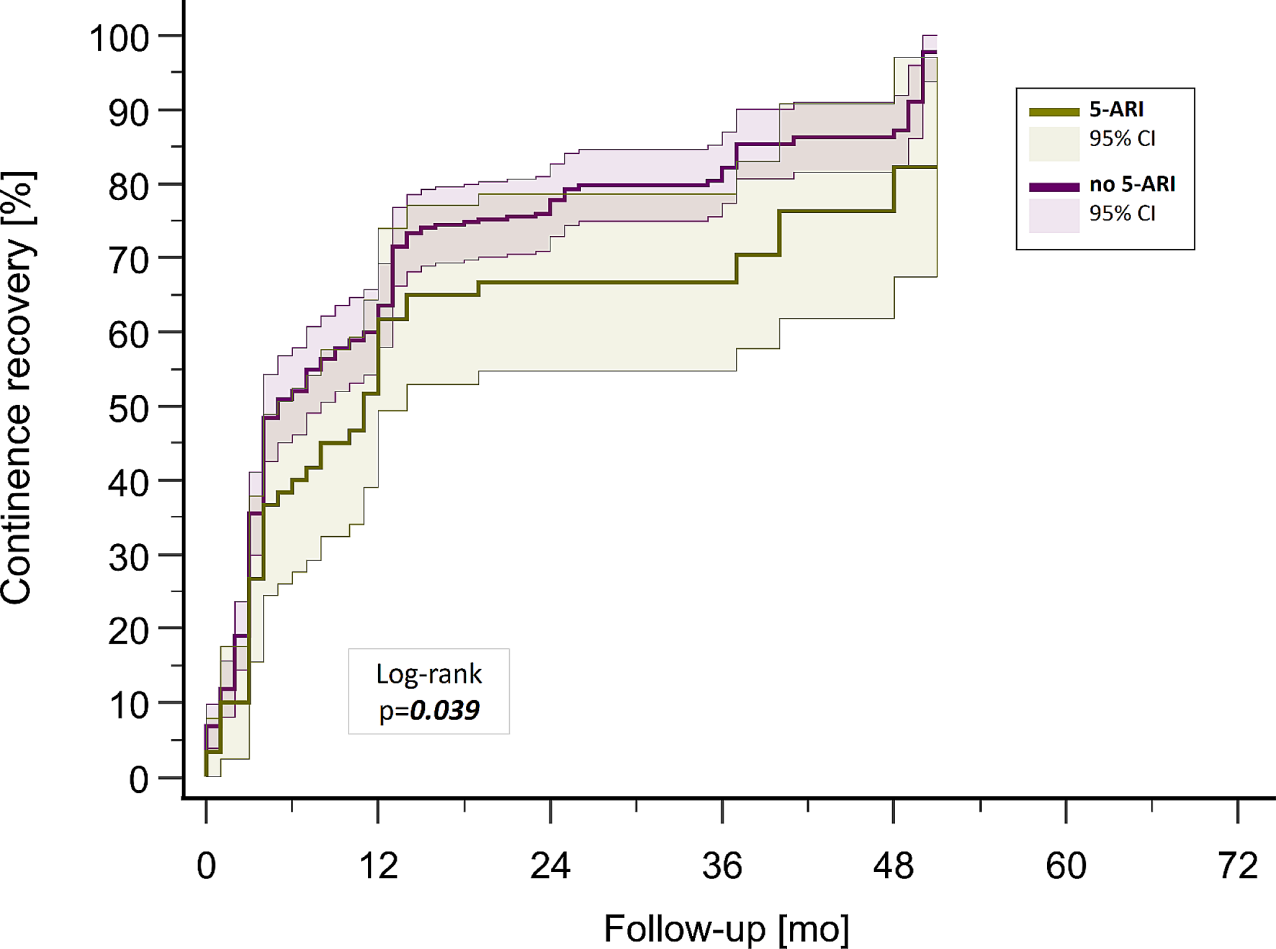
Continence recovery stratified by preoperative 5-ARI usage (CI = confidence interval, mo = months)

Continence recovery probabilities during the follow-up period are displayed in Fig. 2. In multivariable Cox regression analysis stratified for sociodemographic and clinicopathological variables preoperative 5-ARI use was confirmed as an independent predictor of impaired postoperative continence recovery (HR 0.50, 95% CI 0.27–0.94, *p* = 0.03) (Table 2).

**Table 2:** Multivariable cox regression analysis regarding the endpoint continence recovery (5ARI = 5-alpha reductase inhibitor, HR = hazard ratio, bold values indicate *p* < 0.05

Multivariable Cox Regression Analysis				
Impact of 5ARI on Continence recovery				
Parameter	HR	95% CI		*p* value
		**Lower**	**Upper**	
5ARI [y/n]	0.500	0.27	0.94	***0.03***
Age (yrs)	0.907	0.82	1.00	***0.05***
BMI	1.044	0.98	1.11	0.17
Prostate volume	1.010	1.00	1.02	**0.02**
pT-stage	0.916	0.72	1.16	0.47
Nerve-sparing	0.735	0.16	3.39	0.69
Surgical approach	0.718	0.45	1.14	0.16
Preoperative ICIQ-SF-score	0.932	0.85	1.02	0.14
Preoperative IPSS-score	1.022	0.99	1.06	0.19
Subsequent radiotherapy to the prostate	1.122	0.67	1.88	0.66
post RP pelvic floor muscle training	1.062	0.45	2.51	0.89

### Preoperative 5-ARI use and postoperative HRQOL

Pre- and postoperative HRQOL outcomes based on the validated QLQ-C30 as well as QLQ-PR25 questionnaires are summarized in Suppl. Table 2. Briefly, significantly increased global health status scores could be detected for the no-5-ARI subgroup 12 months [34.0 (5-ARI) vs. 47.1 (no 5-ARI), *p* = 0.045] and 24 months [36.4 (5-ARI) vs. 53.4 (5-ARI), *p* = 0.043] postoperatively, indicating better general HRQOL at these time-points for patients without preoperative 5-ARI use (Fig. 1B).

### Preoperative 5-ARI use and postoperative erectile function

Preoperative as well as postoperative comparison of erectile function based on IIEF-5 scores is summarized in Suppl. Table 1.

Preoperatively, mean IIEF-5 score was numerically higher for patients without 5-ARI without reaching statistical significance (8.3 vs. 10.4, *p* = 0.124). Postoperatively, mean IIEF-5 scores were numerically higher for the 5-ARI subgroup throughout the follow-up period without reaching statistical significance. 48 months postoperatively, a significantly higher proportion of patients from the 5-ARI subgroup recovered erectile function, defined as IIEF-5 scores of 18 or higher (26.3 vs. 7%, *p* = 0.017).

### Preoperative 5-ARI use and survival outcomes

Estimated 5-year-biochemical recurrence-free survival (BRFS) rates were 74% for the 5-ARI subgroup compared to 53% for patients without preoperative 5-ARI treatment (*p* = 0.0039; Suppl.Figure 2A). Metastasis-free survival (MFS) based on conventional or PSMA-PET-imaging did not differ significantly between both subgroups with estimated 5-year MFS rates of 91% (5-ARI) vs. 81% (no 5-ARI, *p* = 0.296; Suppl. Figure 2)).

## Discussion

While the impact of treatment with 5-ARI on the risk of cancer-related mortality in men with PCa has been extensively studied, little is known about the impact of preoperative 5-ARI use on patient-reported outcomes following RP.

In the present study, we provide data from a well-balanced PS-matched patient cohort that underwent RP at one tertiary care referral centre with a median follow-up of 45 months.

Oncological impact of concomitant 5-ARI use in the localized PCa setting has been assessed extensively and no safety concern with regards to stage shift, upgrading [4] or cancer-specific mortality has been reported [2] so far. Thus, use of 5-ARI in prostate cancer can be considered safe from an oncological point of view. In line with these findings, our propensity score-matched analysis did not identify any significant differences in 5-year-MFS between the patients with and without preoperative 5-ARI treatment. Interestingly 5-year-BRFS rates were higher for the no-5ARI-cohort. While the rates of positive surgical margins and locoregional lymph node invasion were both higher for the no-5ARI cohort, a potential effect of the 5ARI-treatment on post-RP PSA-values cannot completely be ruled out.

The current analysis is the first to show a significant impact of preoperative 5-ARI use on postoperative mid-term and long-term continence recovery with independent prognostic impact on time to continence recovery. Notably this was assessed using both, the validated ICIQ-SF questionnaire as well as daily pad usage and confirmed in the multivariable Cox regression analysis.

One might argue that preoperative 5-ARI treatment does not have a negative impact on postoperative urinary function per se but rather represents a surrogate for impaired bladder function due to chronic subvesical urinary track obstruction. However, it has to be emphasized that in our matched cohort, preoperative prostate volume, which has been shown to negatively impact urinary function recovery [14, 16], did not significantly differ between both subgroups. In addition, preoperative IPSS-scores were significantly higher in the subcohort without preoperative 5-ARI treatment, indicating moderate lower urinary tract symptoms preoperatively for this subgroup. Thus, a pure surrogate effect of preoperative 5-ARI treatment seems unlikely based on the current data.

Based on current hypotheses, urinary continence recovery post-RP is a complex interplay between pelvic skeletal muscles including the external urinary sphincter, smooth muscle fibers as well as the urethral bulb [17].

Beyond that, the impact of androgens on skeletal muscle functions is well-established [18]. While testosterone induces skeletal muscle hypertrophy through multiple pathways, for instance through modulation of pluripotent mesenchymal cells [15], there is no evidence that preoperative low serum testosterone leads to delayed urinary function recovery post-RP [19]. In addition, it has been shown that 5-ARI do not have a significant effect on serum testosterone levels [20] making a significant pathogenetic impact of testosterone levels in this setting more unlikely.

Physiologically, 5-alpha reductase catalyzes the reduction of testosterone into dihydrotestosterone. Importantly, there is evidence that 5-alpha reductase is expressed on skeletal muscle fibers where it plays a crucial role in myotrophic pathways [21]. For dihydrotestosterone, it has been shown that dihydrotestosterone activates the MAPK [22] as well as Akt/mTOR and GLUT4 pathway [23], leading for instance to increased maximum force of skeletal muscle cells.

Regarding the impact of 5-alpha reductase on smooth muscle fibers, there is preclinical data that suggests that 5-ARI causes epithelial and stromal changes by affecting the intra-prostatic homeostatic interaction between the epithelium and the underlying stroma, ultimately leading to smooth muscle de-differentiation [24]. This could potentially weaken the pelvic floor. Furthermore, the impact of 5-ARI on sexual dysfunction has been previously described and one of the proposed mechanisms focused on reduced levels of nitric oxide due to the lack of dihydrotestosterone [25]. Thus, it can be hypothesized that this lack of nitric oxide negatively affects the corpus spongiosum and therefore the urethral bulb, adding to the aforementioned potentially negative effects of 5-ARI treatment on mid-term and long-term urinary function recovery.

Finally, since long-term survival rates after RP for clinically localized PCa are high [26], HRQOL becomes another essential measure to determine clinical benefit of treatment strategies in those patients. In the current study, we found better general HRQOL outcomes 12 months and 24 months postoperatively for patients without preoperative 5-ARI use and this finding can be adequately explained with the differences in urinary function recovery for these patients. While from an oncological point of view, 5ARI-treatment prior RP appears to be safe, our results show that patients with previous 5ARI therapy require more intensive postoperative care in order to achieve good functional results to allow improved health related quality of life.Our study has several limitations, which are mainly inherent to the retrospective analysis of the prospectively maintained dataset. A potential patient selection bias typical for retrospective analyses can therefore not be completely negated but was aimed to be minimized using the PS-matching method and creating two matched cohorts with similar baseline clinical characteristics that have been shown to impact the postoperative urinary function recovery. However, it has to be acknowledged that detailed information regarding specific indication and exact length of 5ARI-treatment were not available.

## Conclusion

Our findings highlight that preoperative 5-ARI treatment was associated with impaired continence outcomes starting 24 months up to 60 months postoperatively, suggesting that preoperative 5-ARI treatment can impair the long-term urinary function recovery following radical prostatectomy.

## Electronic supplementary material

Below is the link to the electronic supplementary material.

Supplementary Material 1

Supplementary Material 2

Supplementary Material 3

Supplementary Material 4
