# Supplementary material for: The impact of preoperative 5-alpha reductase inhibitors on functional outcomes and health-related quality of life following radical prostatectomy – A propensity score matched longitudinal study
Source: World J Urol. 2024 Jul 22;42(1):432. doi: 10.1007/s00345-024-05108-9 (PMC11263412; doi:10.1007/s00345-024-05108-9)
Supplement: Supplementary file 1 — Supplementary Material 1 [file 345_2024_5108_MOESM1_ESM.docx]

**Suppl. Table 1** Functional outcomes at baseline (T0) and longitudinal follow-up (ICIQ-SF = International Consultation on Urinary Incontinence Questionnaire short-form, IIEF-5 = International Index on Erectile Function, SD = standard deviation). Bold values indicate p<0.05.
