# Supplementary material for: The impact of preoperative 5-alpha reductase inhibitors on functional outcomes and health-related quality of life following radical prostatectomy – A propensity score matched longitudinal study
Source: World J Urol. 2024 Jul 22;42(1):432. doi: 10.1007/s00345-024-05108-9 (PMC11263412; doi:10.1007/s00345-024-05108-9)
Supplement: Supplementary file 2 — Supplementary Material 2 [file 345_2024_5108_MOESM2_ESM.docx]

**Suppl. Table 2**. Patient-reported outcomes at baseline (T0) and longitudinal follow-up (EORTC QLQ = European Organization for Research and Treatment of Cancer quality of life questionnaire, SD = standard deviation). Bold values indicate p<0.05.
