# Supplementary material for: The impact of preoperative 5-alpha reductase inhibitors on functional outcomes and health-related quality of life following radical prostatectomy – A propensity score matched longitudinal study
Source: World J Urol. 2024 Jul 22;42(1):432. doi: 10.1007/s00345-024-05108-9 (PMC11263412; doi:10.1007/s00345-024-05108-9)
Supplement: Supplementary file 3 — Supplementary Material 3 [file 345_2024_5108_MOESM3_ESM.docx]

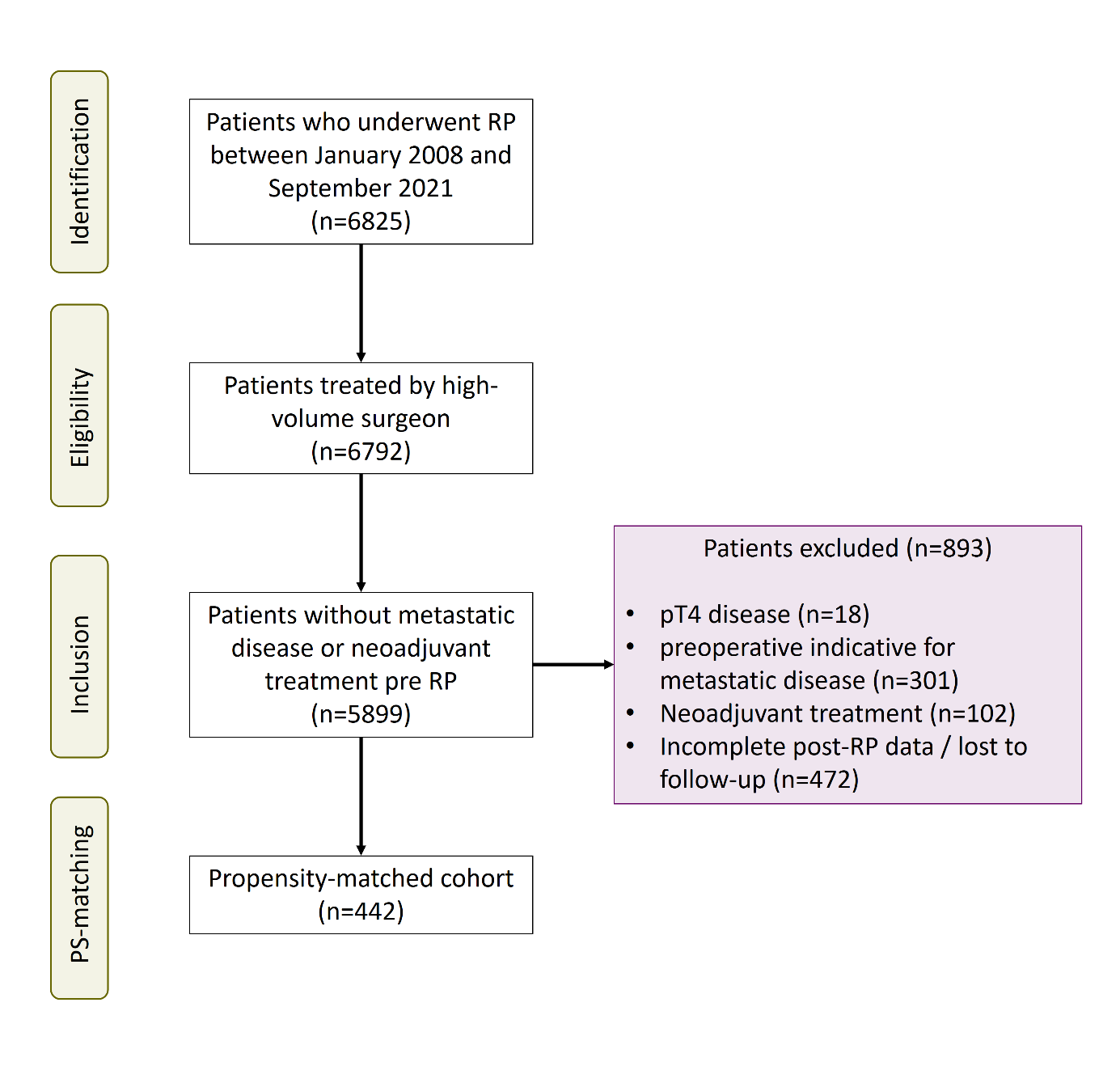


**Suppl. Figure 1.** Flow chart summarizing the inclusion process for eligible subjects including the propensity score (PS) matched cohorts of the current study (RP = radical prostatectomy).
