# Supplementary material for: The impact of preoperative 5-alpha reductase inhibitors on functional outcomes and health-related quality of life following radical prostatectomy – A propensity score matched longitudinal study
Source: World J Urol. 2024 Jul 22;42(1):432. doi: 10.1007/s00345-024-05108-9 (PMC11263412; doi:10.1007/s00345-024-05108-9)
Supplement: Supplementary file 4 — Supplementary Material 4 [file 345_2024_5108_MOESM4_ESM.docx]

**
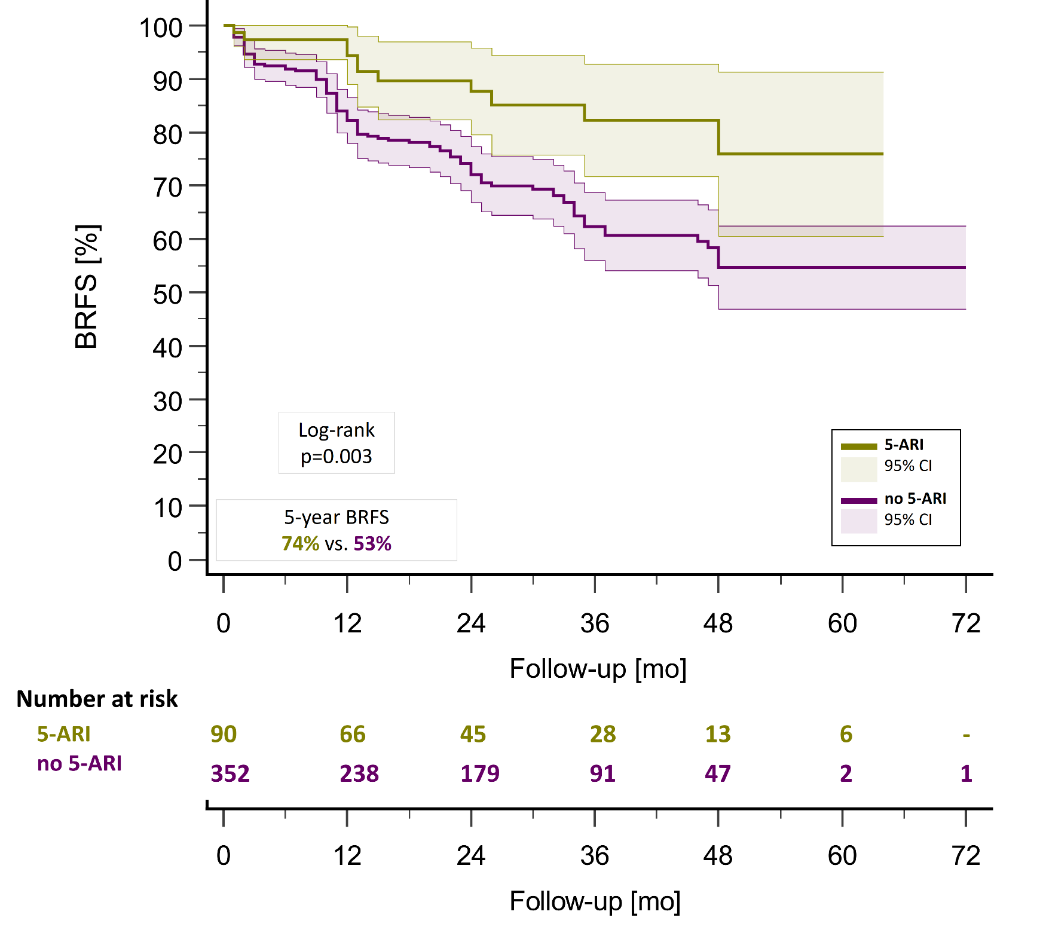
A**

**
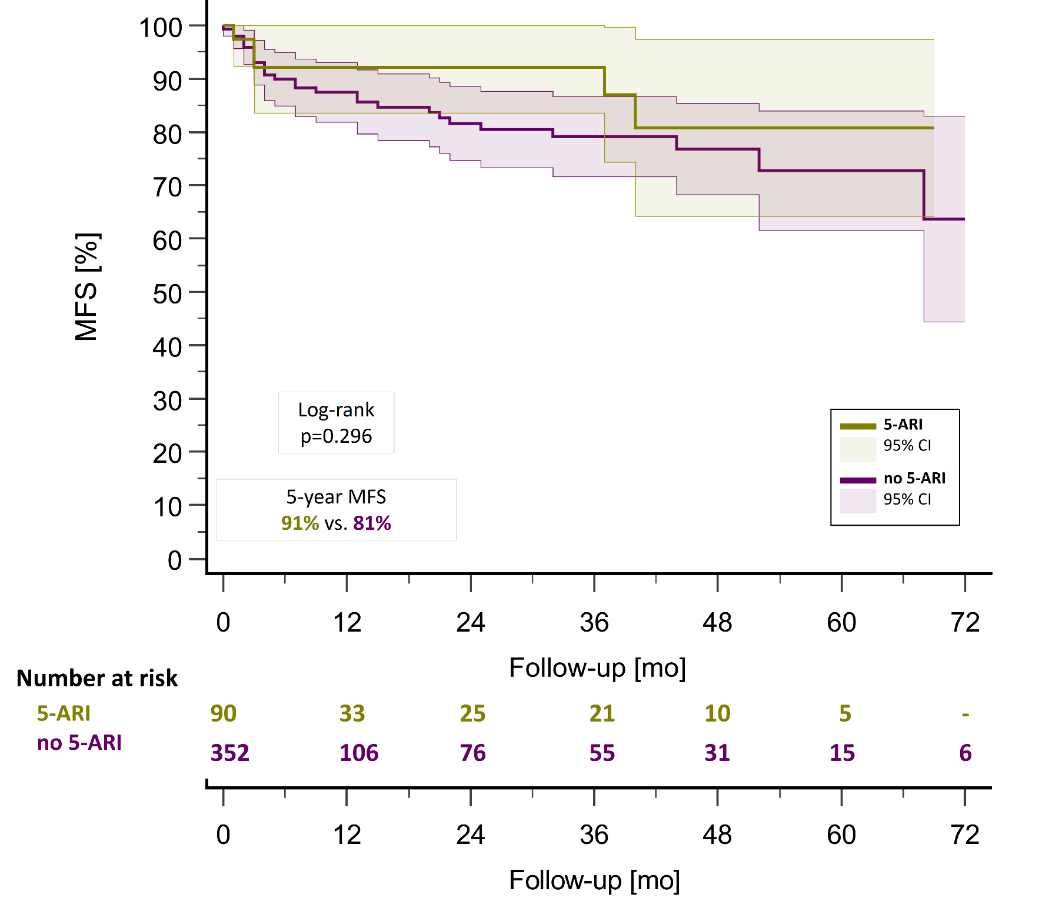
B**

**Suppl. Figure Suppl. Figure 1. (A)** Biochemical recurrence-free survival (BRFS) and **(B)** Metastasis-free survival (MFS) stratified by preoperative 5-ARI usage (CI = confidence interval, mo = months).
